# The impact of role stress and work-family balance on nurses’ professional quality of life: a network analysis

**DOI:** 10.3389/fpsyg.2026.1777395

**Published:** 2026-05-29

**Authors:** Yuecui Kan, Sheng Yang, Xiaoyu Chen, Menghan Xu, Xiaomeng Hu, Bing Li, Jiawei Zhou, Yingying Wang, Ying Xiang, Xiaohui Qiu

**Affiliations:** 1 Psychology and Health Management Center, Harbin Medical University, Harbin, Heilongjiang, China; 2 Department of Psychiatry and Psychology, The Second Affiliated Hospital of Harbin Medical University, Harbin, Heilongjiang, China; 3 The Second Ward of Neurosurgery Department, The First Affiliated Hospital of Harbin Medical University, Harbin, Heilongjiang, China; 4 Department of Endocrinology, The Second Affiliated Hospital of Harbin Medical University, Harbin, Heilongjiang, China

**Keywords:** network analysis, nursing well-being, professional quality of life, role stress, work-family balance

## Abstract

**Objective:**

To explore the network structure linking role stress, work–family balance, and professional quality of life among nurses, and to identify key nodes that may be particularly relevant for future intervention research.

**Methods:**

A cross-sectional survey was conducted with 1,879 nurses from four tertiary hospitals in China. Measures included the Professional Quality of Life Scale, Role Stressors Scale, and Work–Family Balance Scale. A Gaussian Graphical Model with LASSO regularization was estimated to visualize the conditional dependence structure. Strength centrality and bridge strength were computed to identify influential nodes, and stability was assessed via bootstrap procedures.

**Results:**

The network comprised 10 nodes and 39 nonzero edges (86.7% density) across three clusters: professional quality of life, role stress, and work–family balance. The strongest edge within the professional quality of life cluster was between “A1 Compassion Satisfaction” and “A2 Burnout” (weight = 0.64). The strongest cross-cluster edge connected “B3 Role Overload” and “C1 Work-to-Family Conflict” (weight = 0.37). “A3 Secondary Traumatic Stress” showed the highest strength centrality and the highest bridge strength, followed by “C1 Work-to-Family Conflict.” Bootstrap procedures confirmed excellent stability for both strength and bridge strength centrality (CS-coefficients >0.75).

**Conclusion:**

Secondary traumatic stress and work-to-family conflict occupy structurally central and bridging positions in the network linking professional quality of life, role stress, and work–family balance. Their high centrality and bridge strength underscore their potential relevance as targets for future intervention research, although the cross-sectional design precludes causal inference.

## Introduction

Nurses are the backbone of the healthcare system. They often encounter multiple pressures, including heavy nursing tasks, staffing shortages and strict shift work schedules. Professional quality of life is a concept specifically designed to assess the psychological states—both positive and negative—experienced by practitioners in helping professions (such as nurses, doctors, social workers, etc.) during their work. It encompasses both the satisfaction they derive from helping others and the exhaustion and trauma associated with their work (Stamm 2010). Long-term high-intensity work is associated with lower Professional Quality of Life (Hunt et al. 2019). Professional quality of life comprises positive and negative dimensions. The positive aspect, compassion satisfaction, reflects the fulfillment derived from aiding others (Lee et al. 2021), which mitigates occupational stress, enhances work engagement, and improves clinical performance. The negative dimension, compassion fatigue, includes burnout and secondary traumatic stress. Burnout arises from chronic occupational stress, manifesting as emotional exhaustion, depersonalization, and diminished personal accomplishment (Maslach and Jackson 1986). Studies have reported that 11.23% of nurses suffer from job burnout (Woo et al. 2020). Secondary traumatic stress involves trauma-related symptoms (e.g., intrusive memories, hypervigilance) resulting from prolonged patient exposure (Yildirim et al. 2018). The prevalence of secondary traumatic stress is notably high among emergency nurses, with a pooled prevalence of 65% (Xu et al. 2024), and some studies have reported rates as high as 75% in certain regions (Morrison and Joy 2016). Nurses typically report moderate compassion satisfaction and compassion fatigue, with Asian and ICU nurses experiencing more severe compassion fatigue (Xie et al. 2021). A decline in the professional quality of life among nurses not only directly leads to reduced quality of care but also, due to the multiple roles individuals fulfill in society, exerts tangible impacts on nurses’ family lives in practice.

The Conservation of Resources (COR) Theory was proposed by psychologist Hobfoll in 1989 (Hobfoll 1989). Its core premise is that individuals strive to protect and accumulate limited resources when facing stress, as these resources form the foundation for coping with life’s various challenges. Hobfoll posited that an individual’s mental health and well-being are primarily influenced by the acquisition, preservation, and loss of resources. Resources can be categorized into four types: material, social, personal, and emotional resources. When individuals perceive a threat to or loss of their resources, it triggers a stress response, subsequently affecting their psychological state and quality of life. According to COR theory, when nurses encounter multiple role stresses—such as work demands and family responsibilities—they experience resource depletion. Particularly when these stresses are disorganized, excessively frequent, or difficult to regulate, it may be associated with the loss of emotional resources, which in turn corresponds to their professional quality of life. Consequently, understanding the interactions among these variables is crucial for better understanding the professional quality of life among nurses.

To gain a deeper understanding of the professional quality of life among nurses, it is essential to systematically examine the contextual and systemic factors that influence it. The nature of nursing—characterized by heavy workloads, circadian rhythm disruptions, and family obligations—further intensifies work–family conflicts (Barnes-Farrell et al. 2023). Previous studies indicate a close relationship between nurses’ professional quality of life and work-family dynamics. Empirical research demonstrates that work–family conflict is positively correlated with compassion fatigue (including burnout and secondary traumatic stress), suggesting that reducing conflict is associated with lower levels of compassion fatigue (Deisinger et al. 2022; Etesaminia & Tarım, 2025), which relates to professional quality of life. Similarly, effective work-family balance is associated with reduced occupational burnout and secondary traumatic stress, which contributes to the enhancement of compassion satisfaction (Grzywacz and Marks 2000). Work-Family balance refers to the psychological and behavioral state in which individuals achieve coordination, satisfaction, and optimal functioning between their work and family roles (Grzywacz and Marks 2000). Grzywacz and Carlson (2007) conceptualized it as a dynamic process of negotiating role expectations with work and family partners, emphasizing the quality of role performance and interaction satisfaction (Grzywacz and Carlson 2007).

When examining the relationship between nurses’ professional quality of life and work-family interactions, role stress emerges as a critical variable that demonstrates growing significance. The role stress generated by inherent structural and interpersonal demands in clinical environments systematically impacts nurses through three core dimensions: role conflict arising from contradictory expectations (Rizzo et al. 1970), role ambiguity stemming from unclear responsibilities (Rizzo et al. 1970), and role overload caused by excessive task demands (Eatough et al. 2011). This kind of psychological tension and behavioral dysfunction resulted from role stress are particularly prominent in healthcare institutions (Zhang et al. 2024). Previous studies indicate that role conflict and role ambiguity show strong correlations with burnout (Cortese and Ghislieri 2022); and longitudinal data from Chinese nurses further reveal that role ambiguity predicts the development of emotional exhaustion and work alienation (Li et al. 2021). Based on these findings, we can reasonably infer that when nurses assume different roles in the workplace and family domains, role stress may show positive associations with work–family conflict, and this pattern may relate to professional quality of life. However, to date, no studies have examined and integrated the complex relationships among these factors within the nursing population.

Traditional studies on nurses’ professional quality of life have predominantly used regression and structural equation modeling (SEM) to test directional hypotheses among latent or observed variables. While these methods are valuable for examining specific pathways, they often focus on a priori specified relationships and may not fully capture the complex, non-hierarchical, and potentially bidirectional interactions among multiple constructs. Network analysis, grounded in graph theory, offers a complementary perspective by modeling observed variables as nodes and their conditional dependencies as edges using regularized partial correlations. Rather than assuming a latent structure, network analysis conceptualizes psychological constructs as systems of interacting components, enabling the visualization of a dense web of direct relationships without imposing a directional structure (Epskamp et al. 2018; Borsboom and Cramer 2013; Epskamp and Fried 2018a). This approach offers several advantages: it identifies central variables through metrics such as strength, highlights bridge nodes that link different variable communities, and reduces overfitting through techniques like LASSO regularization.

In conclusion, the current study aims to deeply explore the dimensional-level relationship between nurse professional quality of life and its influencing factors according to the Conservation of Resources (COR) Theory through the network analysis method. A Gaussian Graphical Model (GGM) was used to examine the networks among role stress, work–family balance, and professional quality of life. Our goals are to (1) identify key role stress or work–family balance dimensions that are most strongly connected to professional quality of life via centrality analysis, and (2) detect bridge variables facilitating interactions between stress and balance domains. This study is expected to provide guidance for nurses’ mental health and career development, which is of great significance for maintaining the stability of the nursing industry and improving the quality of life of nurses in their profession.

## Methods

### Participants and procedures

This study employed a cluster randomized sampling method, utilizing a combination of online and paper-based questionnaires for data collection. Between January and February 2024, nurses from multiple departments across four first-class tertiary hospitals, randomly selected from eight such institutions, were surveyed. Out of 1,916 initial responses, 1,879 valid questionnaires were retained after excluding invalid data, resulting in a valid response rate of 98%. Data collection utilized four standardized instruments: a general demographic questionnaire, the Professional Quality of Life Scale, the Chinese version of the Work-Family Balance Scale, and the Role Stressors Scale. All participants provided written informed consent prior to questionnaire completion, ensuring data accuracy and strict confidentiality. The study protocol received formal approval from the Institutional Review Board to guarantee research integrity and ethical compliance.

### Measures

Epidemiologic information collected included gender, age, level of education, marital status, number of children, years of employment, and average number of night shifts per month.

Professional quality of life was measured using the 30-item Professional Quality of Life Scale by Stamm (Stamm 2010), comprising three subscales: compassion satisfaction, burnout, and secondary traumatic stress, rated on a 5-point Likert scale. Items 1, 4, 5, 17, and 29 are reverse-scored. Clinical cutoffs are compassion satisfaction <37, burnout >27, and secondary traumatic stress >17, with severity classified as mild (one cutoff), moderate (two), or severe (all three). The scale showed good internal consistency (Cronbach’s *α* = 0.761).

Work–family balance was assessed using the 14-item scale developed by Grzywacz and Marks (Grzywacz and Marks 2000) and adapted into Chinese by Zeng (Zeng and Yan 2013). This instrument conceptualizes work–family dynamics as bidirectional, comprising both conflict and facilitation. Each of these two components contains two unidirectional dimensions: Work-to-Family conflict and Family-to-Work conflict, as well as Work-to-Family facilitation and Family-to-Work facilitation. Items are rated on a five-point scale, with higher scores indicating greater levels of conflict or stronger facilitation. The scale demonstrated acceptable internal consistency in this study (Cronbach’s *α* = 0.722).

Role stress was assessed using the 13-item Role Stressors Questionnaire by Peterson et al. (Peterson et al. 1995), adapted into Chinese by Li et al. (Li and Zhang 2009). It includes three dimensions: role conflict, role ambiguity, and role overload, rated on a 5-point Likert scale. Role ambiguity items are reverse-scored; others are positively scored. Higher scores indicate greater role stress. The scale showed satisfactory internal consistency (Cronbach’s *α* = 0.754).

### Data analysis

Descriptive statistics and correlation analysis were performed using SPSS 27.0. Network analysis was conducted in R software (version 4.4.3) using the qgraph package (Epskamp et al. 2012) to estimate and visualize a Gaussian Graphical Model (GGM). In the GGM, nodes represent observed variables (dimension scores), and edges represent regularized partial correlation coefficients between pairs of nodes after controlling for all other nodes in the network. Partial correlations range from-1 to 1, with blue solid edges indicating positive associations and red dashed edges indicating negative associations; edge thickness is proportional to the absolute value of the partial correlation coefficient.

To obtain a sparse and interpretable network, we applied the Least Absolute Shrinkage and Selection Operator (LASSO)regularization using the Extended Bayesian Information Criterion (EBIC)for model selection, with the hyperparameter gamma(*γ*)set to the default value of 0.5 (Epskamp et al. 2018). All variables were standardized prior to analysis, and no missing data were present in the final dataset (*N* = 1,879).

Centrality was assessed using the bootnet package (Epskamp and Fried 2018b). Recent methodological research has raised concerns about the suitability of betweenness and closeness centrality in psychological networks, as these measures assume the presence of information flow and shortest paths—assumptions that may not align with how psychological variables relate to one another (Bringmann et al. 2019). In contrast, strength centrality, which sums the absolute edge weights connected to a node, directly reflects a node’s local connectivity and has been shown to be more stable and interpretable in psychological networks (Epskamp et al. 2018). Therefore, we focused on strength centrality as the primary centrality metric in this study.

Bridge centrality was computed using the networktools package (Jones 2018) to identify nodes that connect different clusters(professional quality of life, role stress, and work-family balance). Specifically, bridge strength (the sum of absolute edge weights connecting a node to nodes in other clusters) was used as the primary bridge metric.

The stability and accuracy of the network were evaluated via non-parametric bootstrap procedures (1,000 resamples) using the bootnet package (Epskamp and Fried 2018b). Edge weight accuracy was assessed by computing 95%confidence intervals (CIs)for each edge; narrower CIs indicate higher estimation accuracy. Centrality stability was quantified using the correlation stability (CS) coefficient, for which values above 0.5 are considered acceptable and above 0.75 indicate excellent stability (Epskamp et al. 2018).

## Results

### Participant demographic data

In the present study, a total of 1,879 participants met the study inclusion criteria, including 77 males and 1,802 females. The basic socio-demographic characteristics of the participants are detailed in Table 1.

**Table 1:** Participant demographic information (*N* = 1879).

Variables	*n* (%)
Gender	
Men	77 (4.1)
Women	1802 (95.9)
Age	
20–30	252 (13.4)
31–40	1,207 (64.2)
41–50	305 (16.2)
51–60	114 (6.1)
>60	1 (0.1)
Education level	
Vocational school and below	154 (8.2)
Junior college	1,657 (88.2)
College or higher	68 (3.6)
Marital status	
Unmarried	366 (19.5)
Married	1,418 (75.5)
Divorced/separated	95 (5.1)
Number of children	
0	572 (30.4)
1	1,119 (59.6)
2	180 (9.6)
≥3	8 (0.4)
Working years	
1–2	164 (8.7)
3–5	43 (2.3)
6–10	208 (11.1)
>10	1,464 (77.9)
Average number of night shifts per month	
0–4	1,083 (57.6)
5–9	631 (33.6)
≥10	165 (8.8)

### Descriptive statistics and variable correlation analysis

The results of the scores for each dimension of the Professional Quality of Life Scale, Role Stressors Scale, and Work-Family balance are shown in Table 2. The Professional Quality of Life Scale demonstrates the following scores: compassion satisfaction scores was 33.2 ± 3.833 (critical value <37), burnout shows 31.74 ± 5.065 (critical value >27), and secondary traumatic stress measures 25.66 ± 7.343 (critical value >17). The Role Stress Scale reveals: role conflict scores 7.42 ± 3.149, role ambiguity measures 19.2 ± 4.553, and role overload presents 14.81 ± 5.21. On the Work-Family balance Scale, the two highest-scoring dimensions emerge as Work-to-Family conflict (11.19 ± 4.554) and Family-to-Work facilitation (11.01 ± 2.987).

**Table 2:** The score of professional quality of life scale, role stressors scale and work-family balance scale.

Scales	Mean	SD
Professional quality of life scale	90.60	11.456
Compassion satisfaction	33.20	3.833
Burnout	31.74	5.065
Secondary traumatic stress	25.66	7.343
Role stressors scale	41.43	8.851
Role conflict	7.42	3.149
Role ambiguity	19.20	4.553
Role overload	14.81	5.210
Work-family balance Scale		
Work–family conflict	20.52	7.875
Work-to-family conflict	11.19	4.554
Family-to-work conflict	9.34	4.245
Work-family facilitation	20.48	5.344
Work-to-family facilitation	9.47	3.123
Family-to-work facilitation	11.01	2.987

The Pearson correlation was used for each variable in this study, and the results are shown in Table 3. The correlations between the variables were significant (*p* < 0.01), except for the non-significant correlations between Work-family facilitation and Work–family conflict as well as Professional quality of life. Given the large sample size (*N* = 1,879), statistical significance is not the primary focus. Instead, the magnitude of correlations (effect sizes) and, in the network analysis, regularized partial correlation edge weights are emphasized.

**Table 3:** Correlation between professional quality of life, role stress, work–family conflict, and work-family facilitation.

Scales	ProQOL	Role stressors	Work–family conflict	Work-family facilitation
ProQOL	1	–	–	–
Role stress	0.115**	1	–	–
Work–family conflict	0.220**	0.450**	1	–
Work-family facilitation	0.023	0.176**	0.027	1

**p < 0.01.

ProQOL, Professional Quality of Life; Work–family conflict includes Work-to-Family and Family-to-Work conflicts.

Work-family facilitation includes work-to-family and family-to-work facilitation.

### Network analysis

**Figure 1:**
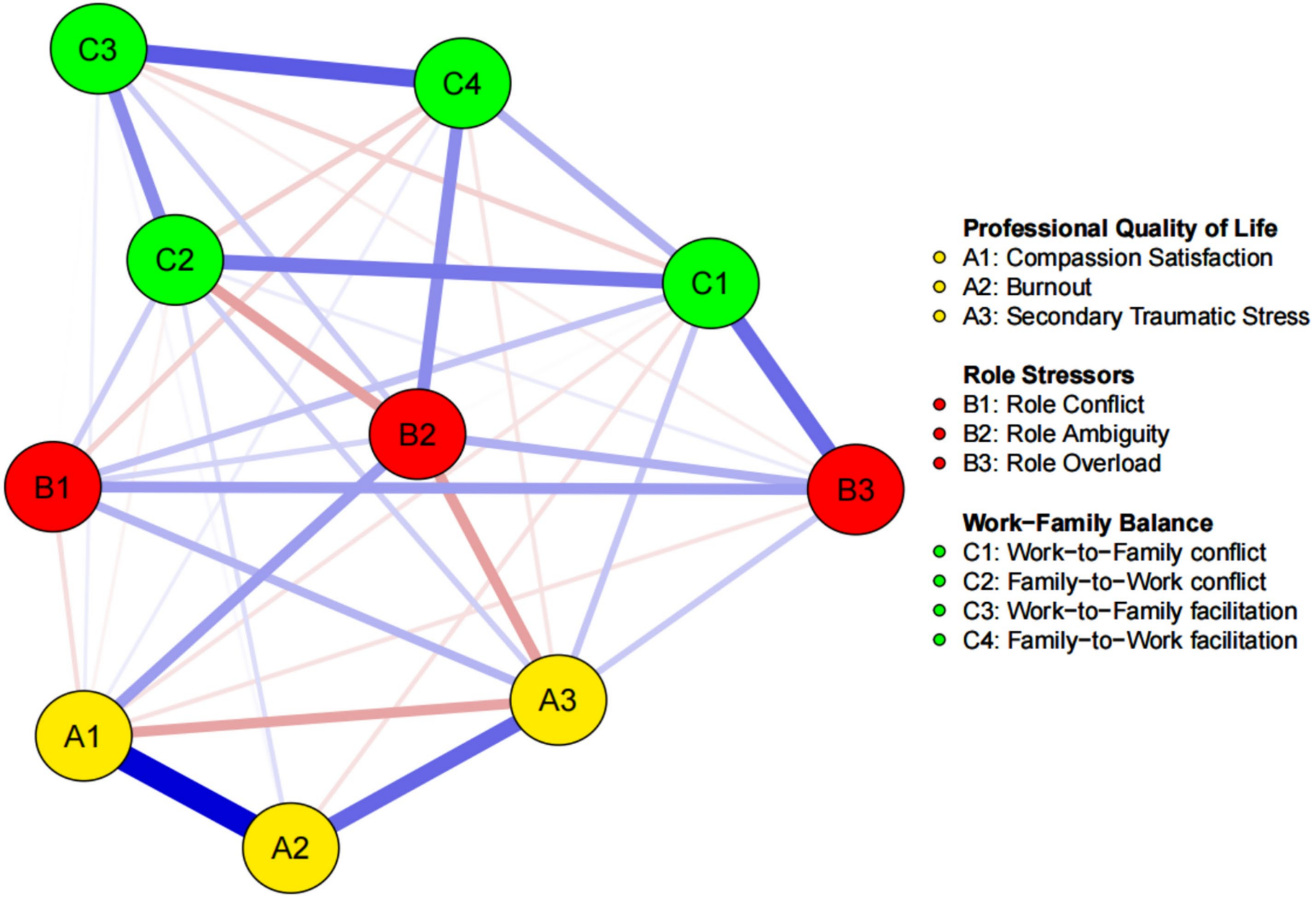
Network of professional quality of life, role stressors, and work-family balance. Nodes represent dimensions, and edges indicate associations between nodes. Edge thickness reflects the strength of the association, while color indicates its polarity (blue for positive, red for negative).

The structure of the network for Professional Quality of Life, Role Stressors, and Work-Family Balance is shown in Figure 1. Ten nodes are clustered into three different clusters, with a total of 39 nonzero edges, including 24 positively correlated and 15 negatively correlated edges. The network structure for occupational quality of life, role stress, and work-family balance is shown in Figure 1.

Within the Professional Quality of Life cluster, the edge with the highest weight occurs between “A1 Compassion Satisfaction” and “A2 Burnout” (weight = 0.64), followed by “A2 Burnout” and “A3 Secondary Traumatic Stress” (weight = 0.39). In contrast, the weight between “A1 Compassion Satisfaction” and “A3 Secondary Traumatic Stress” is −0.23. In the Role Stressors cluster, the weight between “B1 Role Conflict” and “B3 Role Overload” is 0.25. Within the Work-Family Balance cluster, the weight between “C1 Work-to-Family conflict” and “C2 Family-to-Work conflict” is 0.34.

Among the different clusters, the strongest edge between the Professional Quality of Life cluster and the Role Stressors cluster occurs between “A1 Compassion Satisfaction” and “B2 Role Ambiguity” (weight = 0.25), while the strongest edge between the Professional Quality of Life cluster and the Work-Family Balance cluster appears between “A3 Secondary Traumatic Stress” and “C1 Work-to-Family conflict” (weight = 0.14). The strongest edge between the Role Stressors cluster and the Work-Family Balance cluster lies between “B3 Role Overload” and “C1 Work-to-Family conflict” (weight = 0.37).

**Figure 2:**
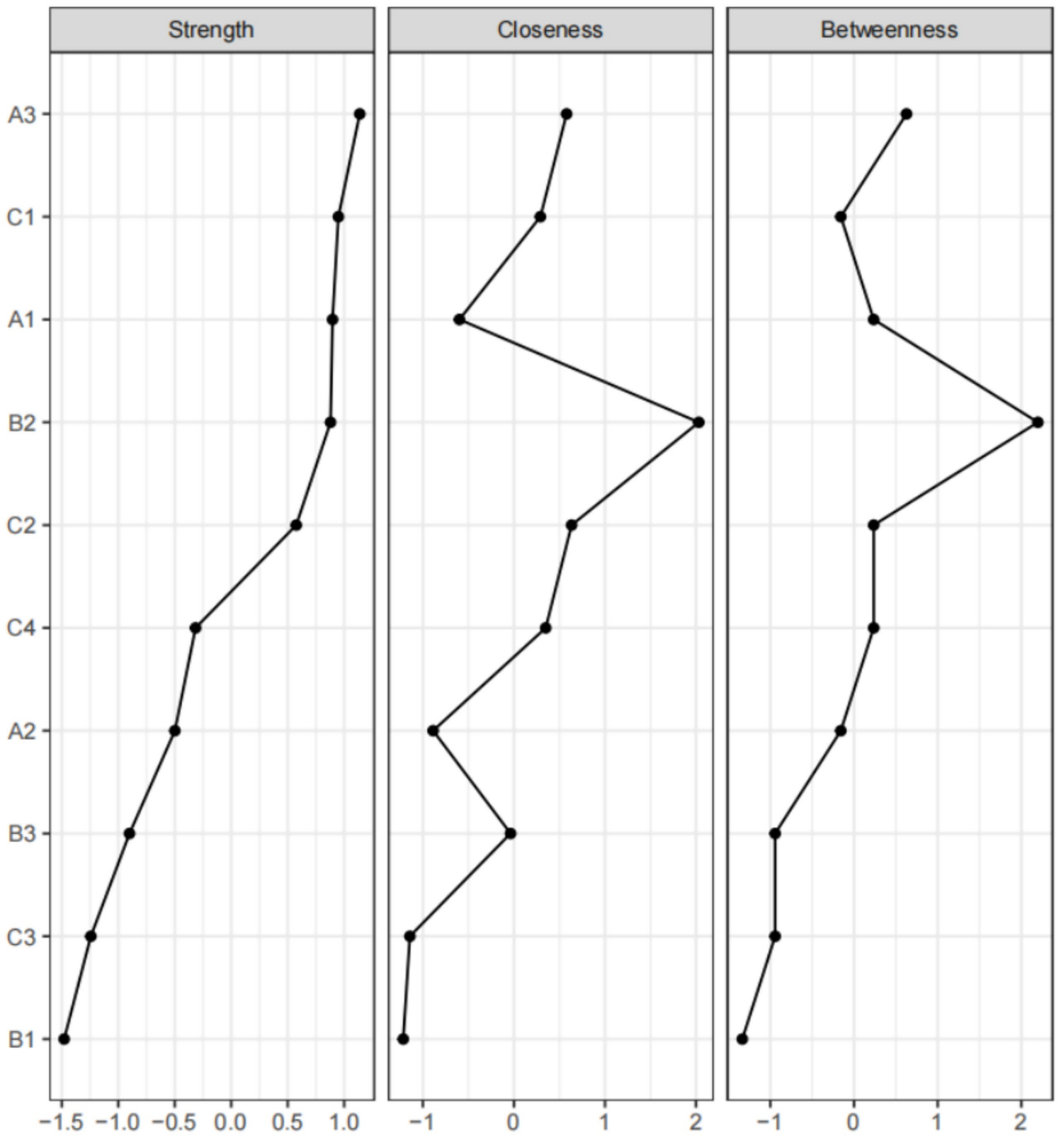
Centrality of all nodes within the network. Greater values indicate greater centrality. The *x*-axis shows the *Z* score.

The centrality indices for the network are shown in Figure 2, sorted by strength centrality for visual comparison. “A3 Secondary Traumatic Stress” had the highest strength centrality, followed by “C1 Work-to-Family conflict,” indicating that these two nodes occupied the most structurally central positions in the network.

**Figure 3:**
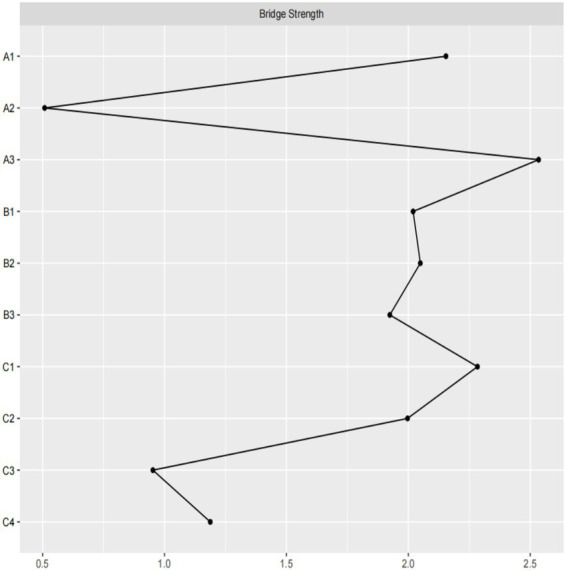
Bridge centrality of all nodes within the network. Greater values indicate greater centrality. The *x*-axis shows the *Z* score.

The bridging centers of the network are shown in Figure 3. The results show that “A3 Secondary Traumatic Stress” is the node with the highest bridge strength, followed by “C1 Work-to-Family conflict”.

**Figure 4:**
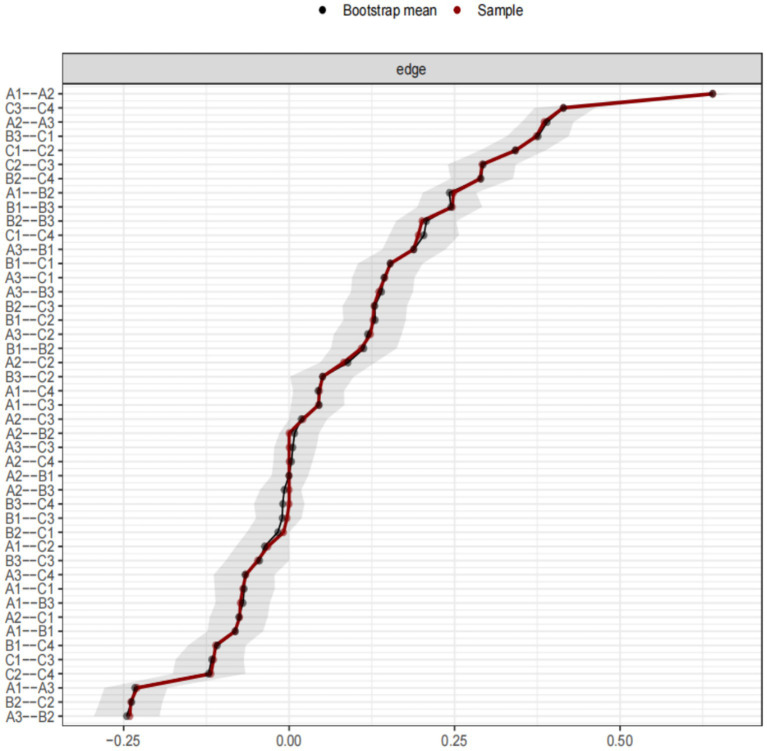
The results of the edge weight bootstrapping procedure.

Figure 4 displays the results of the non-parametric bootstrap procedure (1,000 resamples) for evaluating edge weight accuracy. The x-axis represents edge weight values, and the y-axis lists each edge in the network. Red points indicate the bootstrap mean for each edge, and gray horizontal lines represent the 95% confidence intervals (CIs) from the bootstrap distribution. The 95% CIs for all edges were relatively narrow, and the bootstrap means closely aligned with the sample estimates, indicating high estimation accuracy. The narrow CIs suggest that the estimated edge weights are stable and reliable, supporting the robustness of the network structure.

**Figure 5:**
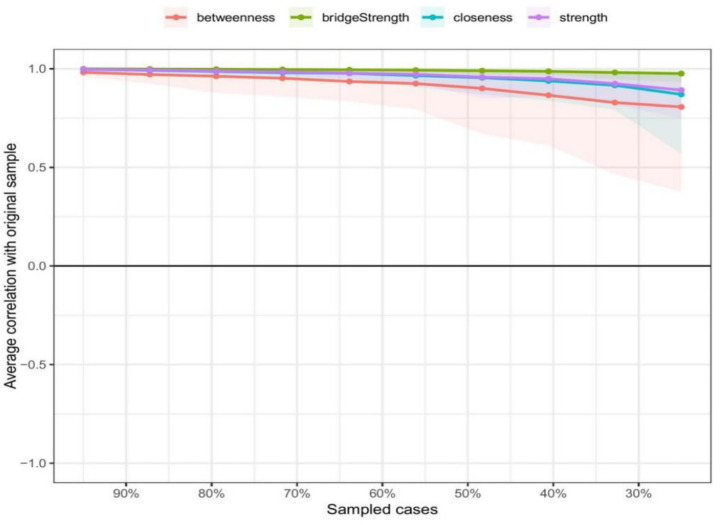
The results of the subset bootstrapping procedure.

Figure 5 displays the case-dropping subset bootstrap results for centrality stability. Strength centrality (CS = 0.85) and bridge strength (CS = 0.90) both exceeded the recommended threshold of 0.75 for excellent stability, confirming that these centrality metrics are reliably estimated and robust to sample composition.

### Cross-hospital consistency

To assess the potential influence of hospital-level clustering, we re-estimated the Gaussian Graphical Model separately for each of the four hospitals (sample sizes: 290, 311, 854, and 424). Pairwise correlations between edge weight vectors ranged from 0.911 to 0.961 (mean *r* = 0.942), indicating that the network structure is largely invariant across hospital settings (see Table 4).

**Table 4:** Pairwise correlations of edge weight matrices across the four hospitals.

Hospital	1	2	3	4
1	1.000			
2	0.952	1.000		
3	0.945	0.961	1.000	
4	0.911	0.928	0.957	1.000

All correlations are Pearson correlation coefficients. Correlations were computed on the vectorized upper-triangular elements of each edge weight matrix.

## Discussion

Descriptive and correlational analyses revealed significant associations among dimensions of professional quality of life, role stress, and work-family balance, with particularly strong correlations between role stress and work–family conflict (*p* < 0.01). Building on these findings, network analysis offered further insights into the interrelations among these constructs. The resulting network included 10 nodes and 39 non-zero edges (24 positive, 15 negative) out of 45 possible edges (86.7%), organized into three clusters: professional quality of life, role stress, and work-family balance. This relatively high density can be understood in light of three factors. First, the large sample (*N* = 1,879) provides high statistical power, which enables the LASSO procedure to retain even small but reliable conditional associations rather than shrinking them to zero. Second, professional quality of life, role stress, and work–family balance are substantively interconnected in the daily experience of nurses, such that a broadly dense pattern of conditional dependencies is theoretically expected. Third, model selection used the default Extended Bayesian Information Criterion (EBIC) tuning parameter *γ* = 0.5, a value shown to perform well across simulation scenarios (Foygel and Drton 2010) and subsequently adopted as the qgraph package default (Epskamp et al. 2018). A sensitivity analysis using a more stringent γ = 0.75 yielded 38 edges (84.4%), with all core edges remaining virtually unchanged. Bootstrap procedures confirmed stable centrality indices (CS-coefficients >0.75) with narrow confidence intervals, indicating that the density reflects genuine complexity rather than a methodological artifact. Within the professional quality of life cluster, the strongest link was between “A1 Compassion Satisfaction” and “A2 Burnout” (weight = 0.64), followed by “A2 Burnout” and “A3 Secondary Traumatic Stress”. Across clusters, the most salient edge connected “B3 Role Overload” and “C1 Work-to-Family conflict” (weight = 0.37). Centrality analysis identified “A3 Secondary Traumatic Stress” and “C1 Work-to-Family conflict” as the most influential nodes, and “A3 Secondary Traumatic Stress” exhibited the highest bridge centrality, linking the three domains. These findings point to nodes that may be particularly relevant for future intervention research.

We first verified the reverse coding of items 1, 4, 5, 17, and 29 of the Professional Quality of Life Scale and confirmed that no scoring errors were present. In the zero-order correlation analysis, “A1 Compassion Satisfaction” and “A2 Burnout” were significantly positively correlated (*r* = 0.623, *p* < 0.001), indicating a moderately strong positive co-variation trend even without controlling for other variables. In the network analysis, after controlling for all other dimensions of role stress and work–family balance, the partial correlation weight between the two was 0.64, making it one of the strongest edges in the entire network. The high consistency in both direction and magnitude between the zero-order correlation and the partial correlation indicates that the positive association between A1 and A2 is not driven by confounding effects of other shared stressors but rather reflects a robust and direct conditional association pattern. This positive association can be understood within the framework of Conservation of Resources (COR) theory. The theory posits that individuals have an innate tendency to obtain, protect, and preserve the resources they value, and when resource depletion is chronic and resource replenishment is insufficient, a resource loss spiral may ensue, which has been linked to negative states such as burnout (Hobfoll 1989). Among nursing populations, the relationship between compassion satisfaction and burnout is more complex than is often assumed. Although a meta-analysis reported an overall negative correlation between the two, the heterogeneity test also revealed significant variability across studies, suggesting that this relationship may vary depending on factors such as work environment and workload (Xie et al. 2021). Evidence from high-demand clinical settings further supports this complexity. For example, in a sample of Chinese nurses, compassion satisfaction, burnout, and secondary traumatic stress were simultaneously elevated, indicating that higher compassion satisfaction can coexist with higher emotional stress in trauma-intensive environments (Li et al. 2022). Among emergency nurses, path analysis results have also shown that compassion satisfaction was positively associated with compassion fatigue (encompassing secondary traumatic stress and emotional overload), which in turn was also positively associated with higher levels of burnout (Yu and Gui 2022). Other research in emergency settings has further found that factors such as empathy, self-compassion, and job satisfaction jointly associated with the network of associations linking compassion satisfaction, burnout, and fatigue, suggesting that strong professional commitment may be related to greater susceptibility to exhaustion under significant organizational stress (Yu et al. 2021). A study conducted in a university hospital also reported that coping strategies such as positive reappraisal were found to moderate the association between compassion satisfaction and burnout, suggesting that the availability of coping resources is related to the strength and direction of the relationship between these two dimensions (Yeşil and Polat 2023). Moreover, a large-scale systematic review found that in high-risk units (e.g., intensive care units, palliative care, and trauma care units), compassion satisfaction, burnout, and secondary traumatic stress often co-occur at elevated levels, further highlighting the contextual and differential role of setting in the relationship between the two (Xie et al. 2021). In summary, the stable positive A1 - A2 relationship observed on the network may reflect a “high input - high consumption” model in the clinical nursing environment of China. Nurses who experience stronger compassion satisfaction also tend to report greater emotional, cognitive, and moral resources in patient care. While such deep engagement is associated with professional meaning, under conditions of chronic understaffing, excessive workload, and cumulative trauma exposure, it may also be associated with higher levels of emotional labor, moral distress, and empathic distress. When these resources are not adequately replenished through organizational or family channels, elevated burnout and high compassion may coexist. Therefore, this robust positive association does not theoretically negate the protective role of compassion; rather, it points to a potential dual characteristic of compassion satisfaction: it is associated with both professional commitment and a sense of purpose, and at the same time, in the absence of adequate resource support, it is associated with greater vulnerability to burnout. This pattern points to the potential value of future intervention efforts that, in addition to focusing on alleviating negative factors, also aim to support the structural and psychological resources that help nurses maintain compassion with lower accompanying burnout.

“A3 Secondary Traumatic Stress” is a node that warrants particular attention due to its prominent position within the network. Specifically, this node is characterized by both high strength centrality and the highest bridge strength, reflecting two distinct yet complementary properties: dense connectivity within the overall network and extensive linkage across different communities. This combined profile indicates that “A3 Secondary Traumatic Stress” is not only highly interconnected with other variables but also occupies a key linking position across the domains of professional quality of life, role stress, and work–family balance.

At the level of specific associations, “A3 Secondary Traumatic Stress” shows a strong positive edge with “A2 Burnout” (weight = 0.39), consistent with prior findings documenting the co-occurrence of trauma-related experiences and emotional exhaustion (Bakker and Demerouti 2017), as well as with the results reported by Ahmad Rayani (Martin et al. 2025). In addition, a negative association is observed between “A3 Secondary Traumatic Stress” and “A1 Compassion Satisfaction” (weight = −0.23), reflecting an inverse co-variation pattern between trauma-related strain and positive professional experiences. This structural configuration corresponds to a pattern in which secondary traumatic stress, reduced compassion satisfaction, and elevated burnout are embedded within the same interconnected subsystem. Furthermore, the negative edge between “A3 Secondary Traumatic Stress” and “B2 Role Ambiguity” (weight = −0.24) indicates that role perception and trauma-related experiences are statistically interrelated within the network structure, consistent with Peng’s findings (Charvin et al. 2025). These variables do not appear as independent constructs but rather as components embedded within a broader configuration of occupational experiences.

Bridge centrality analysis further highlights the cross-domain connectivity of “A3 Secondary Traumatic Stress.” Positive edges between “B3 Role Overload” and “A3 Secondary Traumatic Stress” (weight = 0.14), and between “A3 Secondary Traumatic Stress” and “C1 Work-to-Family conflict” (weight = 0.14), form a linkage pattern spanning multiple communities. This configuration reflects an interconnected cluster in which role overload, trauma-related strain, and work–family conflict co-occur within the same structural system. This pattern is consistent with the Job Demands–Resources (JD–R) framework (Bakker and Demerouti 2007), which conceptualizes job demands, strain-related experiences, and cross-domain functioning as components of an integrated system rather than as isolated or sequential processes. Importantly, within the network framework, these centrality metrics describe structural characteristics of the system and should not be interpreted as indicators of causal influence. Methodological literature in network analysis has noted that nodes with high centrality and bridge strength often receive particular attention in intervention-oriented research. For example, bridge nodes have been discussed in relation to the co-occurrence patterns of undesirable states within networks (Jones et al. 2021), and nodes with high bridge strength have been described as structurally connected to the co-activation patterns of different components of mental health (Gong et al. 2024). Accordingly, such structural indicators are often used to identify nodes of potential relevance for intervention research (Ryan and Hamaker 2022). In this context, prior literature has discussed a range of strategies across organizational, team, and individual levels. At the organizational level, approaches such as optimizing work arrangements (e.g., allocation of high-intensity tasks, workload regulation, and shift system design), strengthening institutional support, and improving the work environment have been associated with more adaptive patterns of occupational functioning. The implementation of Employee Assistance Programs (EAP), including early identification of high-risk staff, provision of professional counseling, and structured post-event debriefing (e.g., within 24–72 h), has also been described as part of institutional support systems (Xu et al. 2021). At the team level, peer support groups have been described as offering opportunities for emotional expression, shared experience, and feedback exchange (Smith et al. 2019), which have been linked to more favorable configurations of professional quality of life. At the individual level, self-compassion training (Charvin et al. 2025), time management strategies (Hu et al. 2025), and resilience-oriented programs (Martin et al. 2025) have shown relevance in related research contexts.

“C1 Work-to-Family conflict” also emerges as a structurally central node within the network. The strong positive association between “B3 Role Overload” and “C1 Work-to-Family conflict” (weight = 0.37) reflects a close statistical linkage between perceived workload and cross-domain strain (Ma et al. 2025). In addition, the positive edge between “C1 Work-to-Family conflict” and “C2 Family-to-Work conflict” (weight = 0.34) indicates that these two forms of conflict tend to co-occur, forming a tightly interconnected subsystem linking occupational and family domains. From a cross-domain perspective, “C1 Work-to-Family conflict” links objective work conditions with subjective psychological experiences. Its associations with higher levels of burnout and lower levels of “C4 Family-to-Work facilitation” (Ma et al. 2025) reflect a structural configuration in which negative and positive aspects of work–family dynamics are inversely embedded within the network. This pattern highlights its relevance as a key node within the overall system. Accordingly, strategies discussed in prior literature include organizational-level approaches (e.g., optimization of scheduling systems and clarification of role boundaries) (Xu et al. 2021), as well as individual-level approaches (e.g., time management and adaptive coping strategies) (Hu et al. 2025). When informed by network structural indicators, such approaches may be examined in relation to overall network organization rather than isolated outcomes.

## Conclusion

In this study, a psychological network analysis was used to explore the complex interactions influencing nurses’ professional quality of life. This finding underscores the relevance of traumatic stress and work-life imbalance as central factors in nurse well-being, which may inform future research or interventions. Other central nodes also demonstrated strong influence and may serve as valuable targets for future prevention and intervention strategies. These findings suggest that closer attention to nurses’ emotional and family-related stressors, and the adoption of flexible, resource-sensitive approaches, may be beneficial for supporting their mental health and professional functioning. Future intervention research is needed to evaluate such strategies.

### Limitations and future prospects

First, while the cross-sectional design can reveal associative networks among variables, it cannot determine causal directionality. Future longitudinal studies are required to examine potential dynamic relationships between nodes. Second, reliance on self-report measures may introduce social desirability bias. Subsequent research should incorporate multimodal validation through physiological markers (e.g., cortisol levels) and behavioral observations. Third, sample representativeness limitations exist, as potential moderators like departmental differences (e.g., ICU vs. general wards) and work tenure were not fully considered in network structure analyses. Finally, as all participants were recruited from tertiary hospitals, caution is warranted when generalizing findings to primary care institutions.

## Data Availability

The raw data supporting the conclusions of this article will be made available by the authors, without undue reservation.
